# Variations in the Mitochondrial Genome of a Goldfish-Like Hybrid [Koi Carp (♀) × Blunt Snout Bream (♂)] Indicate Paternal Leakage

**DOI:** 10.3389/fgene.2020.613520

**Published:** 2021-01-21

**Authors:** Yude Wang, Wenzhen Sun, Qianhong Gu, Jiajun Yao, Huifang Tan, Xu Huang, Qinbo Qin, Min Tao, Chun Zhang, Shaojun Liu

**Affiliations:** State Key Laboratory of Developmental Biology of Freshwater Fish, Engineering Research Center of Polyploid Fish Reproduction and Breeding of the State Education Ministry, College of Life Sciences, Hunan Normal University, Changsha, China

**Keywords:** red crucian carp-like fish lineage, goldfish-like fish lineage, distant hybridization, mitochondrial DNA, variations

## Abstract

Previously, a homodiploid goldfish-like fish (2*n* = 100; GF-L) was spontaneously generated by self-crossing a homodiploid red crucian carp-like fish (2*n* = 100; RCC-L), which was in turn produced via the distant hybridization of female koi carp (*Cyprinus carpio haematopterus*, KOC, 2*n* = 100) and male blunt snout bream (*Megalobrama amblycephala*, BSB, 2*n* = 48). The phenotypes and genotypes of RCC-L and GF-L differed from those of the parental species but were similar to diploid red crucian carp (2*n* = 100; RCC) and goldfish (2*n* = 100; GF), respectively. We sequenced the complete mitochondrial DNAs (mtDNAs) of the KOC, BSB, RCC-L, GF-L, and subsequent generations produced by self-crossing [the self-mating offspring of RCC-L (RCC-L-F_2_) to the self-mating offspring of RCC-L-F_2_ (RCC-L-F_3_) and the self-mating offspring of GF-L (GF-L-F_2_)]. Paternal mtDNA fragments were stably embedded in the mtDNAs of both lineages, forming chimeric DNA fragments. In addition to these chimeras, several nucleotide positions in the RCC-L and GF-L lineages differed from the parental bases, and were instead identical with RCC and GF, respectively. Moreover, RCC-L and GF-L mtDNA organization and nucleotide composition were more similar to those of RCC and GF, respectively, compared to parental mtDNA. Finally, phylogenetic analyses indicated that RCC-L and GF-L clustered with RCC and GF, not with the parental species. The molecular dating time shows that the divergence time of KOC and GF was about 21.26 Mya [95% highest posterior density (HPD): 24.41–16.67 Mya], which fell within the period of recent. The heritable chimeric DNA fragments and mutant loci identified in the mtDNA of the RCC-L and GF-L lineages provided important evidence that hybridizations might lead to changes in the mtDNA and the subsequent generation of new lineages. Our findings also demonstrated for the first time that the paternal mtDNA was transmitted into the mtDNA of homodiploid lineages (RCC-L and GF-L), which provided evidence that paternal DNA plays a role in inherited mtDNA. These evolutionary analyses in mtDNA suggest that GF might have diverged from RCC after RCC diverged from koi carp.

## Background

Although hybridization in plants is better studied than hybridization in animals, animal hybrids may be more common than previously thought (Baack and Rieseberg, 2007). It is believed that hybridization leads to the evolution of new species (Seixas et al., 2018). In the natural environments, hybridization has been frequently observed in several groups of fish, including sharks and some teleosts (Dehal and Boore, 2005). Hybridization may accelerate diversification through adaptive infiltration, possibly even leading to nearly instantaneous speciation (Abbott et al., 2013). Such hybridization events are accompanied by rapid genomic changes, including chromosome recombination, genome amplification, differential gene expression, and gene silencing (Hirsch et al., 2012). Significant genomic changes may generate beneficial new phenotypes and reproductive traits, possibly increasing fertility and adaptability (Baack and Rieseberg, 2007; Mlynarcikova et al., 2016).

The mitochondrial genome (mtDNA), the only extranuclear genetic material in animals, is frequently used in assessments of genetic diversity in fish populations and hybrids. MtDNA is useful due to its low molecular weight, relatively rapid substitution rate, multiple copies, and maternal inheritance pattern (Guo et al., 2006). Indeed, mtDNA generally evolves 5–10 times faster than single-copy nuclear genes, and evolution rates have an obvious difference among mtDNA regions (Wan et al., 2004). These characteristics render mtDNA useful for tracing animal lineages. For example, studies using genetic techniques for forensic wildlife species identification have shown that species (and even populations) can be distinguished by mitochondrial genes, such as cytochrome c oxidase 1 (*COI*), cytochrome b (*cytb*), the control region (D-loop), and 16s ribosomal RNA (rRNA) (Hsieh et al., 2001; Branicki et al., 2003; Ogden and Linacre, 2015). In particular, *COI* is highly distinct among species (Mwale et al., 2015) and is thus considered the standard DNA barcoding gene (Hebert et al., 2003).

Most animal cells include mtDNA, which is important for various biological functions; the mtDNA of fish is circular and 16–20 kb long (Ye et al., 2014). Most animal mtDNA have 37 genes: 13 protein-coding genes, two rRNAs, and 22 transfer RNAs (tRNAs) (He et al., 2008). tRNAs are vital for the translation of proteins encoded by the mtDNA. The mtDNA also has a large control region, which contains the necessary regulatory elements for transcription and replication of *NRF-1*, *GABP*, *PPARS*, *ERRS*, *CREB*, *C-MyC*, and *YY1* (Boore, 1999; Taanman, 1999). The control region of mtDNA tends to be evolutionarily informative due to its variability, including nucleotide substitutions, indels, and tandem repeats. Site mutations and structural rearrangements are not distributed randomly across the mtDNA control region; certain sites and domains are hypervariable (Lee et al., 1995). For example, Avise and Saunders (1984) used polymorphisms in mtDNA to analyze hybridization and introgression among sunfish species (Lepomis, Centrarchidae).

**FIGURE 1:**
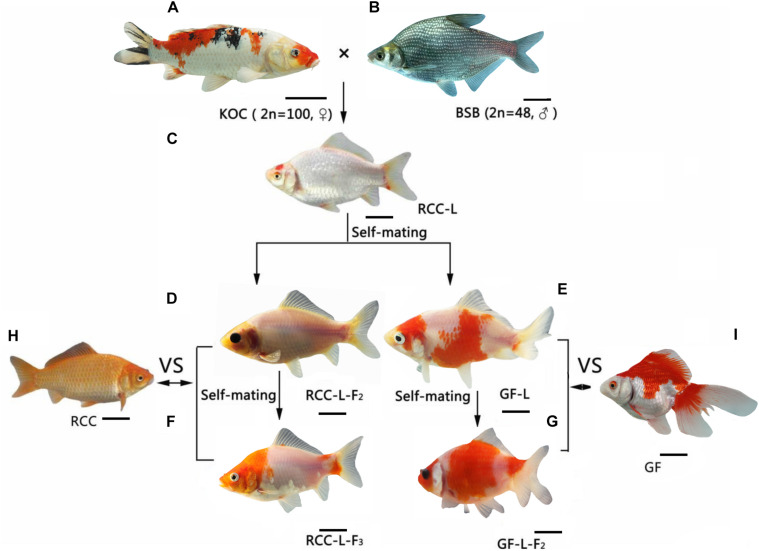
The formation of the hybrid lineages RCC-L (RCC-L-F_1–3_) and GF-L (GF-L-F_1__–__2_) from the distant cross of female koi carp (KOC) and male blunt snout bream (BSB), **(A)** KOC, **(B)** BSB, **(C)** RCC-L, **(D)** RCC-L-F_2_, **(E)** GF-L, **(F)** RCC-L-F_3_, **(G)** GF-L-F_2_, **(H)** RCC, **(I)** GF, Bar = 3 cm.

Although mtDNA recombination is common in most plants, fungi, and protists, this phenomenon was traditionally thought to be rare or absent in animals (Rokas et al., 2003; Xu, 2005). However, variations in mtDNA sequences between animal hybrids and their maternal parents have been reported (Guo et al., 2003), even though mtDNA is usually maternally inherited. In mammals, evidence of paternal inheritance and mtDNA recombination has been found in mice and cattle (Gyllensten et al., 1991; Kaneda et al., 1995). In fish, mtDNA recombination was identified in triploid fish derived from the distant hybridization of female Japanese crucian carp (*Carassius auratus cuvieri*, 2*n* = 100) and male allotetraploid hybrids (4*n* = 200) (Guo et al., 2006). MtDNA recombination was also observed in the F_1_ to F_3_ generations of a crucian carp-like fish lineage that was derived via the distant cross of female common carp (COC, 2*n* = 100) and male blunt snout bream (BSB, 2*n* = 48) (Wang et al., 2019). Koi carp, a variety of common carp that is characterized by red or colorful bodies, have the same age of sexual maturity as blunt snout bream (Wang et al., 2018). Blunt snout bream and koi carp are in the same family (Cyprinidae) but different subfamilies (Cultrinae and Cyprinidae, respectively). Thus, blunt snout bream is an ideal candidate for a cross with koi carp. Previously, we reported that crosses of female koi carp (KOC, 2*n* = 100) and male blunt snout bream spontaneously produced homodiploid red crucian carp-like fish (RCC-L) and homodiploid goldfish-like fish (GF-L) (Figure 1) (Wang et al., 2018, 2020). The phenotypes and genotypes of the RCC-L and GF-L fish differed from those of their parents, but were similar to red crucian carp (RCC) and goldfish (GF) (Wang et al., 2018). However, the evolutionary relationships among koi carp, RCC, and GF have not been well studied.

To address this knowledge gap, we aimed to determine the mtDNA genotypes of the F_1_–F_3_ generations of the RCC-L and GF-L lineages, as well as their parents (koi carp and blunt snout bream), in order to better understand the mitogenomic changes that accompany changes in phenotype and genotype. We also aimed to explore the evolutionary relationships among koi carp, RCC, and GF.

## Materials and Methods

### Experimental Materials

KOC, BSB, three generations of the RCC-L lineage (RCC-L, RCC-L-F_2_, and RCC-L-F_3_), and two generations of the GF-L lineage (GF-L and GF-L-F_2_) were produced via self-mating at the State Key Laboratory of Developmental Biology of Freshwater Fish, Hunan Normal University, China, as described previously (Wang et al., 2018) (Figure 1). All fish were raised in natural pools. All experiments were approved by the Animal Care Committee of Hunan Normal University and followed the guidelines of the Administration of Affairs Concerning Animal Experimentation of China. All dissections were performed after anesthetization with 100 mg/L MS-222 (Sigma–Aldrich, St. Louis, MO, United States), and all efforts were made to minimize suffering.

### DNA Extraction, Amplification, and Sequencing

Total DNA was that extracted from blood samples using UNIQ-10 column genomic DNA extraction kits (Shanghai Biotech). We measured the absorbance of the extracted total DNA at 260 nm using a micro-ultraviolet spectrophotometer (Eppendorf Biophotometer). Extracted DNA was then stored at -20°C.

We designed primers to amplify the full mitogenomes of the RCC-L and GF-L lineages based on the complete mitogenome of the common carp using Primer Premier 5.0 (Ren et al., 2004), Jellyfish 1.4 (Mariani, 2018), and Vector NTI Suite 8 (Lu and Moriyama, 2004) (Supplementary Table 1). Each PCR (50 μL) included 2 ng DNA, 1.5 mmol MgCl_2_, 0.4 μmol forward primer, 0.4 μmol reverse primer, 1× Taq buffer, and 1.25 unit Taq DNA polymerase (Takara). The cycling conditions are given in Guo et al. (2006).

Most of the purified PCR products were sequenced directly by Shanghai Biotech. For fragments that could not be directly sequenced, the purified PCR products were ligated into pMD18-T vectors and then extracted from the positive recombinant plasmids. After PCRs and restriction enzyme digestion, the positive clones were sequenced by Shanghai Biotech.

### Sequence Analysis Across Related Taxa

These sequences were aligned using Blast and ClustalW^1^ and analyzed using MegAlign (version 5.0) and GeneQuest version 7.1 (DNASTAR Software). We determined the lengths of the ETAS, CD, and CBS domains using Blast. The locations of the 13 protein-coding genes in the RCC-L and GF-L sequences were determined by comparing the nucleotide or amino acid sequences with the Mitochondrial Genome Database of Fish^2^. Cloverleaf secondary structures and anti-codon sequences were used to identify the 22 tRNA genes; sequence similarity and secondary structures were used to identify the two rRNA genes (Gutell et al., 1993). In order to know the selectived pressure acted on each protein-coding gene in these species, we calculated the non-synonymous and synonymous substitution rate (dN/dS) using YN00 in PAML software (Yang and Nielsen, 2000) between all the hybrids with their own maternal parents.

### Phylogenetic Analysis

We used the sequences from koi carp, blunt snout bream, RCC, GF, and Danio rerio (Dr), as well as from the RCC-L and GF-L mtDNA. The mtDNA sequences were aligned using ClustalX software (Thompson et al., 2002) and a maximum likelihood (ML) phylogenetic tree was constructed using MEGA 5.0 software (Cummings, 2004). Phylogenetic analysis was performed by ML methods and the best-fitting nucleotide substation model with the lowest BIC score was determined using the Gblocks program (Talavera and Castresana, 2007). ML analyses were performed using the GTR+G, and the robustness of the tree topology was assessed with 1000 bootstrap replicates (Yang and Rannala, 2012).

We estimated that the divergence times using the 16 concatenated gene [13 protein coding gene, two rRNA (12S rRNA and 16S rRNA), and 1 D-loop sequences] by using a lognormal relaxed-molecular-clock (uncorrelated) model implemented in the software package Bayesian Evolutionary Analysis (BEAST, version 1.6.1) (Drummond and Rambaut, 2007). Furthermore, we used the 15 concatenated gene [13 protein coding gene, two rRNA (12S rRNA and 16S rRNA)] estimated the divergence times. The data GenBank number is listed in Supplementary Table 2. The nucleotide substitution models and the model priors for the 16 concatenated gene sequences were gained from the jModelTest analysis above. The fossil calibration points was based on common ancestor of the schizothoracine fish which was in the Oligocene-Miocene boundary (around 23 Ma) (Wang et al., 2016; Wu et al., 2020). Five replicate Markov-chain Monte Carlo (MCMC) analyses were run for 1.0 × 10^8^ generations each, with sampling every 1000 generations. LogCombiner v1.6.1 was used to combine the log files and the resulting trees (Drummond and Rambaut, 2007). BEAST output was analyzed using Tracer v1.7.1^3^, and the appropriate burn-in was determined (100,000 generations). To ensure adequate MCMC mixing, all effective sample sizes (ESS) were >200 for all five runs. We used TreeAnnotator v1.6.1^4^ to summarize the plausible trees and parameter estimates to identify the best-supported tree with maximum clade credibility tree after first discarding the burn-in. We then annotated the summary tree with the mean ages of all nodes and the 95% highest posterior density (HPD) range, as well as the posterior probability at each node (Drummond and Rambaut, 2007). Finally, we used FigTree v1.4.3^5^ to visualize the mean and 95% HPD estimates of divergence times, as well as the posterior probabilities for each of the inferred clades.

## Results

### Genome Organization Across Taxa

**FIGURE 2:**
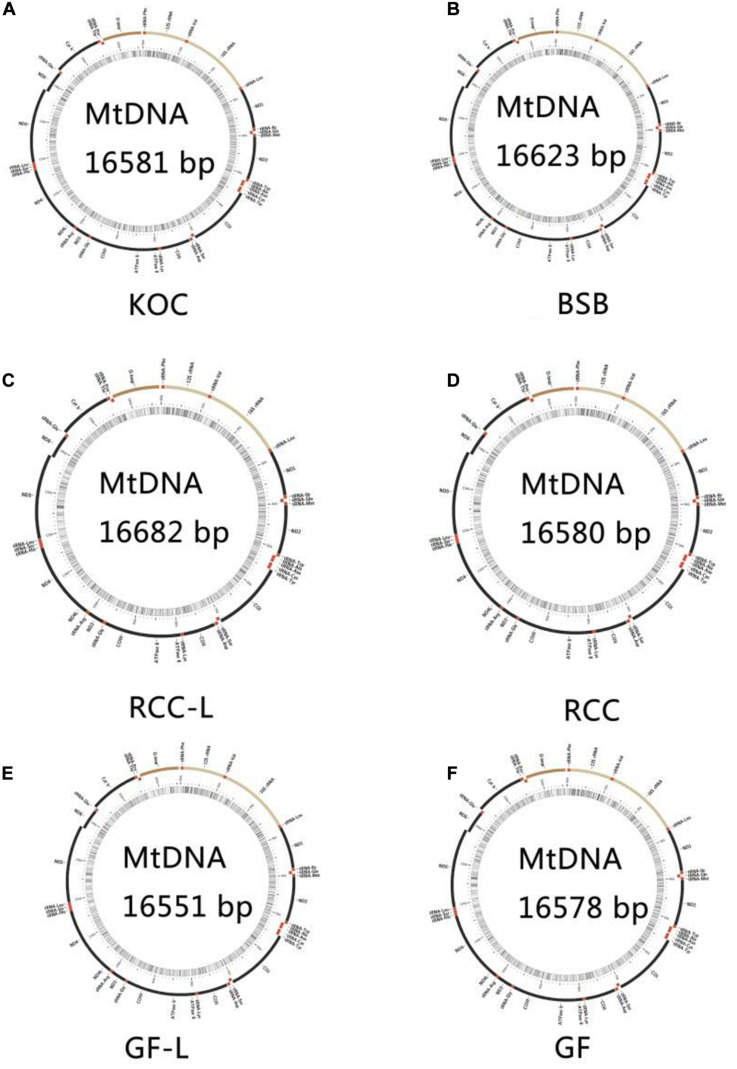
The structure of the mtDNA of **(A)** KOC, **(B)** BSB, **(C)** RCC-L, **(D)** RCC, **(E)** GF-L, and **(F)** GF.

The total lengths of the koi carp, blunt snout bream, RCC-L, GF-L, RCC, and GF mtDNA sequences were 16,581, 16,623, 16,621, 16,551, 16,580, and 16,578 bp, respectively. The mtDNAs of RCC-L and GF-L were similar to those of other published vertebrates: two rRNAs, 22 tRNAs, and 13 protein-coding genes. The lengths of the RCC-L mtDNA (16,621 bp) and the GF-L mtDNA (16,551 bp) were similar to the previously published mtDNA sequences of RCC (16,580 bp) and GF (16,578 bp), respectively. RCC-L and GF-L were more similar in length to koi carp than to blunt snout bream (Figure 2).

The mtDNA control regions of RCC-L and GF-L were 959 and 924 bp in length, respectively, while the lengths of the control regions of koi carp, blunt snout bream, RCC, and GF were 927, 937, 924, and 934 bp, respectively, based on previously published mtDNA sequences. These lengths are typical of fish mtDNA control regions (Ren et al., 2004). Across all sequences, the extended terminal associated sequences (ETAS) domain was 73–75 bp long, the central domain (CD) was 63 bp long, and the conserved sequence block (CSB) domain was 58 bp long.

**FIGURE 3:**
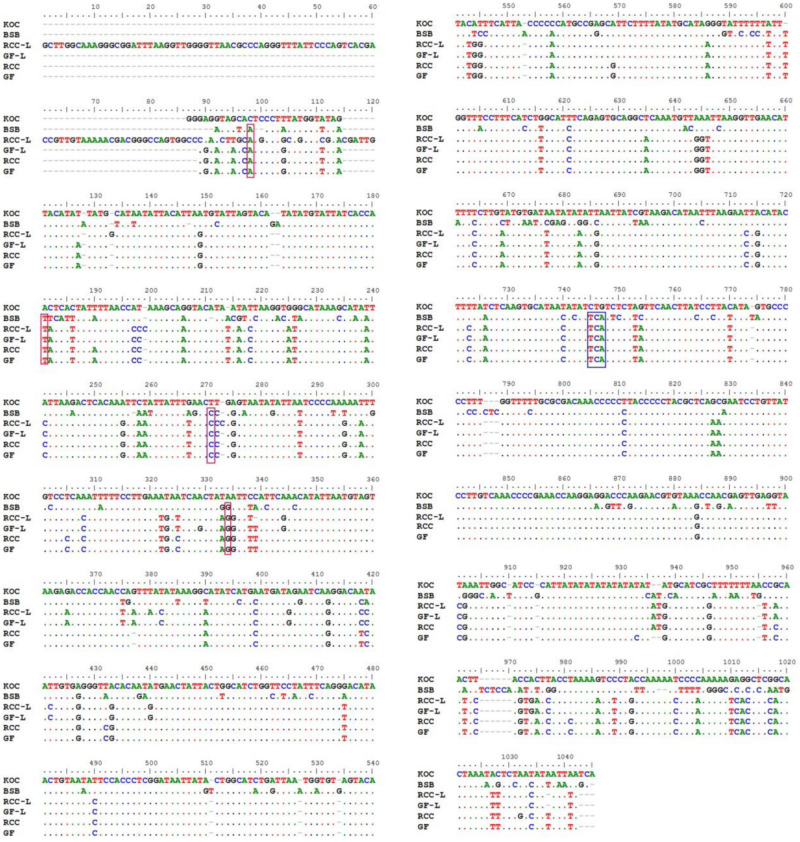
The alignment of the control regions of KOC, BSB, RCC-L, GF-L, RCC, and GF. Dots represent conserved bases, red boxes indicate single-base insertions, and the blue rectangular box indicates the insertion of genetic material from BSB.

There were noticeable differences in base composition across the entire control region and in each domain across the four species, RCC-L, and GF-L (Table 1). With the exception of GF-L, A was the most common base across all taxa, followed by T, C, and G; the mtDNA is known to have low level of base G (Yan et al., 2010). Across all taxa (koi carp, blunt snout bream, RCC, GF, RCC-L, and GF-L), 275 out of 908 (30.29%) nucleotide positions in the mtDNA control region were variable and 245 out of 908 (26.98%) were polymorphic. In addition, 663 out of 1045 (63.44%) were invariable (Figure 3). We found that 12S rRNA was longer in blunt snout bream (962 bp) than in GF-L (954 bp), koi carp (955 bp), RCC-L (955 bp), and RCC (954 bp). Similarly, 16S rRNA was longer in blunt snout bream (1693 bp) than in GF-L (1682 bp), koi carp (1679 bp), RCC-L (1684 bp), and RCC (1682 bp).

**TABLE 1:** Average base composition (%) of the complete mtDNA control region, as well as the three domains within the control region, in KOC, BSB, RCC-L, GF-L, RCC, and GF.

	Entire control region				Extended terminal associated sequences				Central conserved block				Conserved block			
	T	C	A	G	T	C	A	G	T	C	A	G	T	C	A	G
KOC	33.0	19.7	33.3	13.9	39.7	16.4	38.4	5.5	22.2	20.6	38.1	19.1	20.7	34.5	37.9	6.9
BSB	30.5	21.8	33.3	14.4	41.3	13.3	38.7	6.7	25.4	20.6	31.8	22.2	15.5	36.2	39.7	8.6
RCC-L	31.8	21.5	32.7	14.0	41.9	14.9	35.1	8.1	23.8	20.6	34.9	20.6	15.5	36.2	39.7	8.6
GF-L	32.7	20.5	32.4	14.5	41.1	15.1	37.0	6.9	23.8	20.6	34.9	20.6	15.5	36.2	39.7	8.6
RCC	32.5	20.8	32.5	14.2	39.7	15.1	38.3	6.9	23.8	20.6	34.9	20.6	15.5	36.2	39.7	8.6
GF	32.3	20.9	32.5	14.2	39.7	15.1	38.4	6.9	23.8	20.6	34.9	20.6	15.5	36.2	39.7	8.6

Here, we identified 12 identical gene interval sequences and four identical overlapping regions between GF-L and RCC-L; nine identical gene interval sequences and three identical overlapping regions between GF-L and koi carp; and five identical gene interval sequences and one identical overlapping region between GF-L and blunt snout bream. We identified 14,609 conserved and 1972 variable nucleotide sites between GF-L and koi carp; 14,086 conserved and 2537 variable sites between GF-L and blunt snout bream; 16,546 conserved and 78 variable sites between GF-L and RCC-L; 16,325 conserved and 220 variable nucleotide sites between GF-L and GF; and 16,348 conserved and 230 variable nucleotide sites between RCC-L and RCC. Heterogeneity analysis indicated that GF-L and koi carp were 89.70% homologous, with a divergence rate of 11.00%; koi carp and blunt snout bream were 84.6% homologous, with a divergence rate of 16.8%; GF-L and RCC-L were 99.1% homologous, with a divergence rate of 0.5%; GF-L and GF were 87.6% homologous, with a divergence rate of 1.8%; and RCC-L and RCC were 87.0% homologous, with a divergence rate of 0.2%; GF and RCC were 99.9% homologous, with a divergence rate of 0.1%.

### Comparison of Control Regions Across Taxa

Pairwise mtDNA control region sequence identity across the species (koi carp, blunt snout bream, RCC, and GF), as well as RCC-L and GF-L, was 68.2–99.5% (Table 2). Across all pairs, blunt snout bream and RCC-L had the lowest control-region sequence similarity (68.2%), while RCC and GF had the highest (99.5%; Table 2). Although RCC-L was derived from female koi carp, the similarity of control-region sequence was only 77.4% between these two taxa; the similarity of control-region sequence was 86.0% between koi carp and GF-L (Table 2). Importantly, the similarity of control region sequence was 87.0% between RCC-L and RCC, and 97.6% between GF-L and GF (Table 2). Genetic distance, which based on pairwise mtDNA control region sequence comparisons, was lowest between RCC-L and RCC (0.2%), and highest between GF and blunt snout bream (26.4%; Table 2). In terms of RCC-L lineage and GF-L lineage, RCC-L and RCC-L-F_2_ were 94.8% homologous, with a divergence rate of 1.1%; RCC-L-F_2_ and RCC-L-F_3_ were 99.1% homologous, with a divergence rate of 1.4%; RCC-L and RCC-L-F_3_ were 99.4% homologous, with a divergence rate of 0.4%; GF-L and GF-L-F_2_ were 99.5% homologous, with a divergence rate of 0.1%.

**TABLE 2:** Nucleotide sequence similarity (below the diagonal) and divergence (above the diagonal) of the mtDNA control regions of KOC, BSB, RCC-L, GF-L, RCC, and GF.

	KOC	BSB	RCC-L	GF-L	RCC	GF
KOC		24.3%	13.9%	14.3%	13.9%	15.2%
BSB	76.5%		25.0%	25.6%	24.9%	26.4%
RCC-L	77.4%	68.2%		2.0%	0.2%	3.6%
GF-L	86.0%	75.9%	88.8%		2.0%	1.8%
RCC	86.3%	76.3%	87.0%	97.8%		3.6%
GF	86.5%	76.1%	86.8%	97.6%	99.5%	

Across the mtDNA control region, 11.05% of all positions in RCC-L (106/959) and 12.83% of all positions in GF-L (118/920) were inherited from the maternal parent (koi carp). Conversely, 4.38% of all positions in RCC-L (42/959) and 4.35% of all positions in GF-L (40/920) were inherited from the paternal parent (blunt snout bream); 18.25% of all positions in RCC-L (175/959) have mutated, 8.26% of all positions in GF-L (76/920) have mutated, 66.32% of all positions in RCC-L (636/959) in agreement with KOC and BSB; 74.57% of all positions in GF-L (686/920) in line with KOC and BSB. These results revealed that the mtDNA was instable in homoploid generations recently which derived from distant hybridization.

### Comparison of Protein-Coding Genes Across Taxa

Nucleotide composition accurately reflects the basic characteristics of genetic variation among mtDNA sequences. The total lengths of the 13 protein-coding genes of GF-L, RCC-L, koi carp, blunt snout bream, RCC, and GF were 11,373, 11,409, 11,418, 11,409, 11,382, and 11,382 bp, respectively. The similarity of gene sequence between GF-L and RCC-L ranged from 92.5% for *COI* to 100% for *ATPase8* (Table 3). The similarity of gene sequence are ranged from 76.4 to 87.8% between GF-L and koi carp, and from 91.4 to 100% between GF-L and RCC (Table 3). The similarity of gene sequence was substantially greater between GF-L and koi carp than between GF-L and blunt snout bream; conversely, gene sequence variation was lower between GF-L and koi carp than between GF-L and blunt snout bream (Table 3).

**TABLE 3:** Pairwise comparisons of mutation rates and homologies among nucleotide sequences encoding mitochondrial proteins in GF-L, RCC-L, KOC, BSB, RCC, and GF.

Gene	GF-L and RCC-L		GF-L and KOC		GF-L and BSB		RCC-L and RCC		GF-L and GF	
	Divergence (%)	Similarity (%)	Divergence (%)	Similarity (%)	Divergence (%)	Similarity (%)	Divergence (%)	Similarity (%)	Divergence (%)	Similarity (%)
*ND1*	0.1	99.9	12.9	88.3	20.6	82.4	2.4	97.6	1.8	97.8
*ND2*	0.2	99.8	16.2	85.7	22.6	80.8	3.8	96.4	2.2	97.8
*COI*	0.0	100.0	13.2	88.1	16.2	85.7	1.2	98.8	1.2	98.6
*COII*	0.4	99.6	11.2	89.7	13.5	87.8	0.7	99.3	0.0	100.0
*ATPase8*	0.0	100.0	3.8	95.8	20.4	82.4	0.0	100.0	0.0	98.2
*ATPase6*	0.3	99.7	14.2	87.1	20.8	82.1	1.6	98.2	1.6	98.1
*COIII*	0.1	99.9	9.6	90.1	15.0	86.6	1.3	98.7	0.8	99.0
*ND3*	0.3	99.7	16.3	85.7	22.8	80.8	1.2	98.9	1.2	98.6
*ND4L*	0.3	99.7	16.7	85.5	18.5	83.8	1.0	99.0	1.4	97.6
*ND4*	0.1	99.9	15.9	86.0	19.4	83.1	0.0	100.0	1.6	98.3
*ND5*	1.4	98.6	14.4	87.2	24.1	79.3	1.8	98.2	2.0	97.9
*ND6*	0.6	92.5	15.1	80.7	20.7	76.4	1.6	98.5	0.0	100.0
*cytb*	0.6	99.4	12.7	88.5	18.9	83.3	2.3	97.7	2.1	97.8

Like other vertebrate mtDNA (Talavera and Castresana, 2007; Yang and Rannala, 2012), most variable sites resulting in synonymous mutations were at the third codon position. The non-synonymous/synonymous rate ratio (dN/dS) is widely used as an indicator of selection pressure at the sequence level among different species. It is generally believed that dN > dS, dN = dS, and dN < dS indicate positive selection, neutral mutation, and negative selection, respectively (Guo et al., 2003). In this study, all dN/dS values of most protein coding genes were less than 1 in F_1_ hybrids and F_2_ hybrids with its own female maternal parent (Table 4), which indicates that these genes are under negative selection. Among the 13 protein coding genes, the highest dN/dS value was detected from the atp8 gene (0.0480) in the five groups (RCC-L-KOC, RCC-L-F_2_-KOC, RCC-L-F_3_-KOC, GF-L-KOC, and GF-L-F_2_-KOC) (Table 4). Compared with synonymous substitutions, non-synonymous substitutions are more strongly affected by natural selection. And fixations of slightly deleterious mutations are expected to increase the non-synonymous substitution rate (Drummond and Rambaut, 2007). There may be a fixation of slightly deleterious mutations, which leads to an increase of non-synonymous substitutions in F_1_ hybrids and F_2_ hybrids. The increase of mutation rate may be associated with morphological variation.

**TABLE 4:** The dN/dS values between the F_1_ hybrids, F_2_ hybrids, F_3_ hybrids, and their own female maternal parents (KOC).

Gene	RCC-L-KOC	RCC-L-F_2_-KOC	RCC-L-F_3_-KOC	GF-L-KOC	GF-L-F_2_-KOC
Nd1	0.0059	0.0059	0.0044	0.0044	0.0044
Nd2	0.0270	0.0270	0.0299	0.0263	0.0247
Cox1	0.0079	0.0079	0.0056	0.0068	0.0068
Cox2	0.0140	0.0140	0.0086	0.0131	0.0096
Atp8	0.0480	0.0480	0.0480	0.0480	0.0480
Atp6	0.0050	0.0050	0.0077	0.0050	0.0050
Cox3	0.0078	0.0078	0.0010	0.0010	0.0010
Nd3	0.0039	0.0039	0.0040	0.0039	0.0039
Nd4L	0.0041	0.0041	0.0041	0.0048	0.0041
Nd4	0.0056	0.0056	0.0056	0.0066	0.0056
Nd5	0.0226	0.0226	0.0221	0.0248	0.0219
Nd6	0.0072	0.0072	0.0078	0.0081	0.0078
cytb	0.0147	0.0147	0.0072	0.0136	0.0136

### Comparison of rRNA and tRNA Genes Across Taxa

Pairwise comparisons indicated that the sequence similarities for the two rRNA genes (*12S* and *16S*) across all taxa were 91.5–99.8 and 91.3–99.8%, respectively (Supplementary Table 3).

The *12S* rRNA gene sequence similarity was 97.5% between GF-L and koi carp; 91.5% between GF-L and blunt snout bream; 99.8% between GF-L and RCC-L; 99.4% between GF-L and GF; and 99.5% between RCC-L and RCC. The *16S* rRNA gene sequence similarity was 94.8% between GF-L and koi carp; 91.3% between GF-L and blunt snout bream; 99.8% between GF-L and RCC-L; 99.2% between RCC-L and RCC; and 99.3% between GF-L and GF.

Pairwise comparisons indicated that the sequence similarities for the 22 tRNA genes across all taxa were 44.9–99.2% (Supplementary Table 3). In particular, the average tRNA gene sequence similarity was 99.2% between GF-L and RCC-L, as well as between GF-L and GF. In contrast, the average tRNA gene sequence similarity was 45.7, 44.9, and 45.9% between GF-L and koi carp, GF-L and blunt snout bream, and RCC-L and RCC, respectively.

### Genetic Variation Within the RCC-L and GF-L Lineages

Previously, we found that the *5S* rDNA gene was stably inherited by subsequent generations after continuous self-crossing. Here, RCC-L and GF-L were self-crossed to produce RCC-L-F_2_, RCC-L-F_3_, and GF-L-F_2_ (Figure 1); the complete mtDNAs of these offspring were submitted to NCBI (Supplementary Table 2). The lengths of the mtDNA sequences of RCC-L-F_2_ and RCC-L-F_3_ were 16,621 and 16,621 bp, respectively, while that of GF-L-F_2_ was 16,576 bp. MtDNA sequence similarity was high across both lineages. For example, the mtDNA sequence similarity was 99.8% between RCC-L and RCC-L-F_2_; 99.8% between RCC-L and RCC-L-F_3_; 99.9% between RCC-L-F_2_ and RCC-L-F_3_; and 99.6% between GF-L and GF-L-F_2_. RCC-L and RCC-L-F_2_ were 99.5% homologous, with a divergence rate of 0.2%; RCC-L-F_2_ and RCC-L-F_3_ were 99.1% homologous, with a divergence rate of 0.6%; RCC-L and RCC-L-F_3_ were 98.6% homologous, with a divergence rate of 0.7%; and GF-L and GF-L-F_2_ were 99.6% homologous, with a divergence rate of 0.1%.

### Phylogenetic Relationships Among Taxa

**FIGURE 4:**
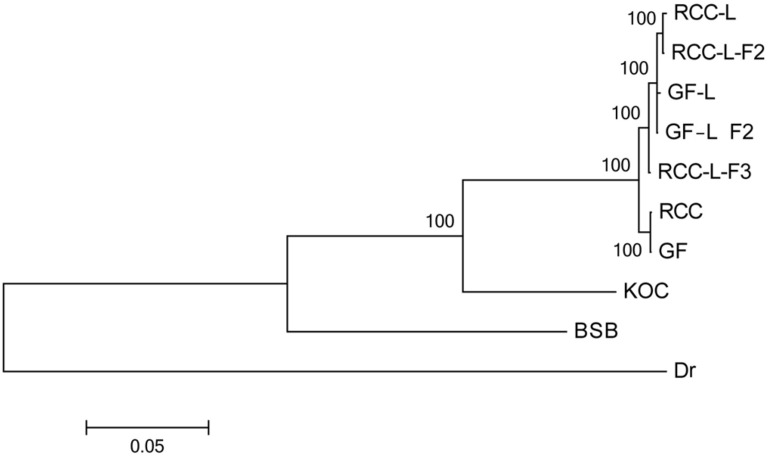
Phylogenetic relationships among KOC, BSB, RCC-L, RCC-L-F_2_, RCC-L-F_3_, GF-L, GF-L-F_2_, RCC, GF, and Danio rerio based on entire mtDNA.

Our phylogeny indicated that RCC-L and GF-L formed a clade, distinct from the clade of RCC and GF (Figure 4). A larger clade, including these taxa as well as koi carp, was sister to the clade including blunt snout bream (Figure 4). Across the taxa analyzed in this study (koi carp, blunt snout bream, RCC, GF, RCC-L, and GF-L), genetic distance was smallest between RCC-L and GF, and largest between GF-L and blunt snout bream. Unsurprisingly, the genetic distance between GF and koi carp was less than that between GF and blunt snout bream. Our phylogeny suggested that RCC-L, GF-L, RCC, and GF were descended from the same ancestor again. Furthermore, we validated this ML tree by constructing an ML tree with IQ-tree (Nguyen et al., 2015) (Supplementary Figure 1).

**FIGURE 5:**
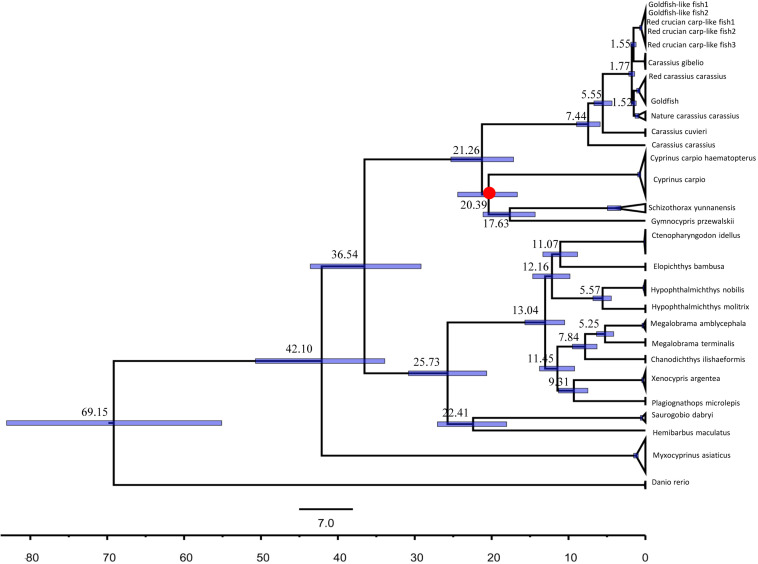
Dated Bayesian phylogeny of KOC, BSB, RCC-L, GF-L, RCC, and GF, as well as other fish in the Cyprinidae (e.g., *Carassius carassius*, *Carassius gibelio*, *Carassius auratus*, and *Carassius cuvieri*), based on the concatenated mitochondrial genes including 13 protein coding genes, two rRNA, and one Doop. Schizothoracine fish calibration points were used to estimate the time of divergence between the GF-L lineage and GF.

Our fossil-calibrated phylogenetic trees indicated that the clade containing koi carp and the clade containing blunt snout bream diverged approximately 42.10 Mya [95% HPD: 50.72–33.91 Mya] (Figure 5). Our results also suggested that RCC diverged from RCC-L approximately 1.77 Mya [95% HPD: 2.16–1.40 Mya] (Figure 5). Similar to our ML analysis, RCC and GF formed a clade, as did RCC-L and GF-L; these pairs appeared to have diverged very recently (Figure 5). Our phylogenies suggested that RCC-L, GF-L, RCC, and GF descended from the same ancestor. Furthermore, we validated this ML tree by constructing an ML tree with IQ-tree (Nguyen et al., 2015) (Supplementary Figure 1). There was no significant difference (*P* > 0.05) between the divergence times using the 16 concatenated gene (Figure 5) and that using the 15 concatenated gene (Supplementary Figure 2).

## Discussion

Hybridization, a major driver of bio diversification and speciation, has been reported in approximately 25% of all known plant species and 10% of all known animal species (Lu and Moriyama, 2004; Mariani, 2018). Similar to introgression, hybridization introduces novel genetic diversity and may be particularly important for speciation (Apte et al., 2003). Although instances of hybrid speciation without corresponding changes in ploidy (i.e., homoploid hybrid speciation) have yet to be reported, homologous hybrid species can be relatively easily identified using genetic techniques (Gutell et al., 1993; Yang and Nielsen, 2000; Thompson et al., 2002; Cummings, 2004). Previously, we obtained a diploid GF-L fish with twin tails (2*n* = 100) by self-crossing an RCC-L fish, where RCC-L was itself derived from the distant crossing of female koi carp and male blunt snout bream (Wang et al., 2018). Our previous analyses of microsatellite pattern (SSR) and *5S* rDNA sequences indicated that GF-L and RCC-L were genetically similar to GF and RCC, respectively (Talavera and Castresana, 2007).

Our genetic structure analysis indicated that the species most similar to GF-L was RCC, followed by koi carp and blunt snout bream (Supplementary Tables 4, 5). The basic composition of the protein coding genes in GF-L was ≥ 92.5% similar to that of RCC-L; in addition, the base composition of GF-L was more similar to koi carp than to blunt snout bream. Codon usage in the GF-L protein-coding genes was very similar to that in GF, while codon usage in the RCC-L protein-coding genes was very similar that in RCC. In both GF-L and RCC-L, codon usage was more similar to koi carp than blunt snout bream. Heterogeneity analysis indicated that GF-L was most similar to GF, and least similar to blunt snout bream. Thus, our results indicated that the mtDNA structures of GF-L were consistent with the expected pattern of maternal inheritance, although some gene intervals and overlapping regions inherited from the blunt snout bream were identified (Supplementary Tables 4–6).

Previous studies have reported instances of mtDNA recombination (Drummond and Rambaut, 2007; Yang and Rannala, 2012). Indeed, Awadalla et al. (Wang et al., 2016) showed extensive recombination in primate mtDNA genes using linkage imbalance analysis, and suggested that the recombined mtDNA derived from both maternal and paternal mtDNAs. Here, we wished to determine whether the GF-L loci that differed from maternal mtDNA, but was identical to the paternal loci, was the result of mtDNA recombination between the paternal and maternal genomes.

Other studies of mtDNA have shown that base insertions and deletions in the control and spacer regions are frequent; these polymorphisms also occur in tRNA and rRNA, but the frequency is low. In addition, the length of the tRNA was significantly different between RCC-L and koi carp, as well as between RCC and blunt snout bream. This result suggested that the mtDNA of RCC-L has undergone a dynamic process.

GF-L and RCC-L, which both have 100 chromosomes (2*n* = 100), are phenotypically similar to GF and RCC, respectively. As the appearance of GF-L is highly dissimilar to that of koi carp or blunt snout bream, it may be inferred that mitochondrial structures differ among these taxa. Microsatellite experiments have identified large amounts of blunt snout bream genetic material in the DNA of GF-L and RCC-L, which may be consistent with the significant structural changes found in RCC-L and GF-L. The phylogenetic analyses showed that two groups (RCC and RCC-L; GF and GF-L) had low genetic diversity. The Molecular clock suggested the KOC and RCC derived the differentiation of the same ancestor at about 21.26 Mya [95% HPD: 16.67–24.41 Mya].

The high degree of mtDNA sequence similarity between RCC-L and RCC-L-F_3_, as well as between GF-L and GF-L-F_2_, suggested that the mitogenomes of both lineages were stably inherited by subsequent generations after continuous self-crossing. Thus, the RCC-L and GF-L lineages may represent an excellent system within which to study the relationship between morphological variation and genetic change, as well as the evolution of novel vertebrate phenotypes.

## Data Availability Statement

The datasets presented in this study can be found in online repositories. The names of the repository/repositories and accession number(s) can be found in the article/Supplementary Material.

## Ethics Statement

The animal study was reviewed and approved by all experiments were approved by the Animal Care Committee of Hunan Normal University and followed the guidelines of the Administration of Affairs Concerning Animal Experimentation of China.

## Author Contributions

SL conceived and designed this study. YW and QG contributed to experimental work, most statistical analyses, and the manuscript writing. QQ, HT, WS, and XH contributed to primers design and bioinformatics analyses. YW and JY collected experimental materials. MT and CZ collected photographs. All authors have read and approved the final manuscript.

## Conflict of Interest

The authors declare that the research was conducted in the absence of any commercial or financial relationships that could be construed as a potential conflict of interest.

## Footnotes

1 http://www.ebi.ac.uk/clustalw

2 http://mitofish.aori.u-tokyo.ac.jp/annotation/input.html

3 http://beast.bio.ed.ac.uk/LogCombiner

4 http://beast.bio.ed.ac.uk/TreeAnnotator

5 https://www.softpedia.com/progChangelog/FigTree-AR-Changelog-172863.html

## Abbreviations

GF-L: the first generation of Goldfish-like fish
GF-L-F_2_: the self-mating offspring of GF-L
MtDNA: mitochondrial DNA
RCC-L: the first generation red crucian carp-like fish
RCC-L-F_2_: the self-mating offspring of RCC-L
RCC-L-F_3_: the self-mating offspring of RCC-L-F_2_.
