# Supplementary material for: Variations in the Mitochondrial Genome of a Goldfish-Like Hybrid [Koi Carp (♀) × Blunt Snout Bream (♂)] Indicate Paternal Leakage
Source: Front Genet. 2021 Jan 21;11:613520. doi: 10.3389/fgene.2020.613520 (PMC7861200; doi:10.3389/fgene.2020.613520)
Supplement: Supplementary file 1 [file Data_Sheet_1.docx]

**Supplementable Table 1** PCR primers for mitochondrial amplification

| Primer | Sequences (5’–3’) |
| --- | --- |
| Mtd1F: GGCTCCCAAAGCCAGA ATTCT | Mtd1R: GCCCTCTTAACACTAAC TGAGT |
| Mtd2F: CACCCCCAAGGGAATT CAGCAG | Mtd2R: GGTACTATTTCTATTGC TTTGTG |
| Mtd3F: CCTAACACTAAAAATT AAATCAT | Mtd3R: TGCTGGCAAATAGAAT TATTGC |
| Mtd4F: GTAGAAGCAACAACAA AATACTT | Mtd4R: CGTAATCTGTATACTCA TAGCTT |
| Mtd5F: GACCCCCACCTAACAA TCAAAG | Mtd5R: AGACTGCTGCTAATACT ATTGAT |
| Mtd6F: CTACCAAAAGCACATG TAGAAGC | Mtd6R: CAAGGATTAGCAGTGC TTGCTA |
| Mtd7F: GGAGTTAAAATCTCCTT ACTCA | Mtd7R: ACCAATCGTAAAATAA GTTGAG |
| Mtd8F: CCGAAAAATAGGAGGC CTAT | Mtd8R: AGAAAATAGTTTAGTTT AGAA |

**Supplementable Table 2** The GenBank number used in the article

| **Species** | **GenBank accession number** |
| --- | --- |
| *Myxocyprinus asiaticus* | AY526869 |
|  | NC_006401 |
|  | AB223007 |
|  | AP006764 |
|  | AY986503 |
| *Carassius carassius* | JQ911695 |
| Goldfish | KU146528 |
| *Megalobrama amblycephala* | EU434747 |
|  | MF522177 |
| Red *carassius auratus* | KJ1874428 |
|  | KJ874430 |
|  | AY714387 |
| Improved red *carassius_auratus* | KX781320 |
| Cyprinus carpio | X61010 |
|  | KU050703 |
|  | MH202953 |
| Goldfish-like fish | MK726307 |
|  | Mk726306 |
| Elopichthys bambusa | NC_024834 |
|  | KM196112 |
|  | NC_024834 |
| Hemibarbus maculatus | JF906109 |
| Carassius cuvieri | AB045144 |
|  | AP011237 |
| Cyprinus carpio haematopterus | JX188254 |
|  | KP993138 |
|  | JN105354 |
| Schizothorax yunnanensis | KR780749 |
|  | KP892531 |
| Gymnocypris przewalskii | KT833113 |
| Hypophthalmichthys molitrix | EU315941 |
|  | NC_010156 |
| Chanodichthys ilishaeformis | NC_029722 |
|  | KU200257 |
| Red curcian carp-like fish | MK658748 |
|  | MK726305 |
|  | MK688992 |
| Saurogobio dabryi | KF612272 |
|  | KU314696 |
| Megalobrama terminalis | NC_018816 |
|  | AB626850 |
| Common diploid crucian carp | GU086395 |
| Common tetraploid crucian carp | GU086397 |
| Plagiognathops microlepis | NC_022711 |
|  | KF383387 |
| Xenocypris argentea | AP009059 |
|  | AP011283 |
|  | NC_008682 |
| Hypophthalmichthys nobilis | EU343733 |
|  | HM162839 |
|  | NC_010194 |
| Danio rerio | NC_002333 |
|  | KM244705 |
| Carassius gibelio | GU138989 |
|  | GU170401 |
|  | KX505166 |
| Ctenopharyngodon idellus | KT894100 |
|  | MG827396 |
|  | EU391390 |
|  | NC_010288 |

**Supplementable Table 3** Comparison of the mutation rate and similarity between the nucleotide sequences of GF-L, RCC-L, Koi, BSB, RCC and GF in 2rRNA and 22 tRNA genes

| **Gene** | **Length (bp)** | | | | | **GF-L and RCC-L** | | **GF-L and KOC** | | **GF-L and BSB** | | **RCC-L and RCC** | | **GF-L and GF** | |
| --- | --- | --- | --- | --- | --- | --- | --- | --- | --- | --- | --- | --- | --- | --- | --- |
|  | **GF-L** | **RCC-L** | **KOC** | **BSB** | **RCC** | **Divergence (%)** | **Similarity (%)** | **Divergence (%)** | **Similarity (%)** | **Divergence (%)** | **Similarity (%)** | **Divergence (%)** | **Similarity (%)** | **Divergence (%)** | **Similarity (%)** |
| 12s rRNA | 954 | 955 | 955 | 962 | 954 | 0.1 | 99.8 | 2.5 | 97.5 | 8.0 | 91.5 | 0.4 | 99.5 | 0.6 | 99.4 |
| 16s rRNA | 1682 | 1684 | 1679 | 1693 | 1682 | 0.1 | 99.8 | 5.1 | 94.8 | 8.1 | 91.3 | 0.7 | 99.2 | 0.6 | 99.3 |
| tRNA-Phe | 69 | 69 | 69 | 69 | 69 | 0.0 | 100.0 | 4.5 | 95.7 | 11.0 | 89.9 | 0.0 | 100.0 | 1.5 | 98.6 |
| tRNA-Val | 72 | 72 | 72 | 72 | 72 | 0.0 | 100.0 | 0.0 | 100.0 | 2.9 | 97.2 | 1.4 | 98.6 | 0.0 | 100.0 |
| tRNA-Leu-1 | 76 | 76 | 76 | 76 | 76 | 0.0 | 100.0 | 1.3 | 98.7 | 2.7 | 97.4 | 0.0 | 100.0 | 0.0 | 100.0 |
| tRNA-Ile | 72 | 72 | 72 | 72 | 72 | 0.0 | 100.0 | 1.4 | 98.6 | 22.0 | 81.9 | 0.0 | 100.0 | 0.0 | 100.0 |
| tRNA-Gln | 71 | 71 | 71 | 71 | 71 | 0.0 | 100.0 | 1.4 | 98.6 | 12.8 | 88.7 | 1.4 | 98.6 | 1.4 | 98.6 |
| tRNA-Met | 69 | 69 | 69 | 69 | 69 | 0.0 | 100.0 | 0.0 | 100.0 | 7.7 | 92.8 | 1.5 | 98.6 | 1.5 | 98.6 |
| tRNA-Trp | 71 | 71 | 71 | 71 | 71 | 1.4 | 98.6 | 4.4 | 95.8 | 6.0 | 94.4 | 1.4 | 98.6 | 0.0 | 100.0 |
| tRNA-Ala | 69 | 69 | 69 | 69 | 69 | 1.5 | 98.6 | 11.2 | 89.9 | 22.9 | 81.2 | 0 | 100.0 | 1.5 | 98.6 |
| tRNA-Asn | 73 | 73 | 73 | 73 | 73 | 0.0 | 98.6 | 2.9 | 94.5 | 0.0 | 100.0 | 0.0 | 98.6 | 0.0 | 100.0 |
| tRNA-Cys | 69 | 69 | 67 | 68 | 69 | 0.0 | 100.0 | 4.7 | 92.8 | 11.4 | 88.4 | 0.0 | 100.0 | 0.0 | 100.0 |
| tRNA-Tyr | 71 | 71 | 71 | 71 | 71 | 0.0 | 100.0 | 2.9 | 97.2 | 7.6 | 93.7 | 0.0 | 100.0 | 0.0 | 100.0 |
| tRNA-Ser-1 | 71 | 71 | 71 | 71 | 71 | 0.0 | 100.0 | 7.7 | 92.8 | 7.6 | 93.0 | 0.0 | 100.0 | 0.0 | 100.0 |
| tRNA-Asp | 72 | 72 | 72 | 74 | 72 | 0.0 | 100.0 | 5.8 | 94.4 | 9.0 | 89.2 | 0.0 | 100.0 | 0.0 | 100.0 |
| tRNA-Lys | 76 | 76 | 76 | 76 | 76 | 0.0 | 100.0 | 0.0 | 100.0 | 9.8 | 90.8 | 0.0 | 100.0 | 0.0 | 100.0 |
| tRNA-Gly | 72 | 72 | 72 | 72 | 72 | 0.0 | 100.0 | 1.4 | 98.6 | 5.8 | 94.4 | 0.0 | 100.0 | 0.0 | 100.0 |
| tRNA-Arg | 70 | 70 | 70 | 70 | 70 | 0.0 | 100.0 | 6.1 | 94.3 | 12.8 | 88.6 | 0.0 | 100.0 | 0.0 | 100.0 |
| tRNA-His | 69 | 69 | 69 | 69 | 69 | 0.0 | 100.0 | 11.3 | 89.9 | 4.5 | 95.7 | 3.0 | 97.1 | 3.0 | 97.1 |
| tRNA-Ser-2 | 70 | 70 | 69 | 69 | 69 | 1.5 | 98.6 | 7.7 | 92.8 | 24.8 | 79.7 | 7.7 | 91.4 | 0.0 | 100.0 |
| tRNA-Leu-2 | 73 | 73 | 73 | 73 | 73 | 0.0 | 100.0 | 1.4 | 98.6 | 7.4 | 93.2 | 0.0 | 100.0 | 4.5 | 95.7 |
| tRNA-Glu | 69 | 69 | 69 | 69 | 69 | 0.0 | 100.0 | 0.0 | 100.0 | 6.1 | 94.2 | 0.0 | 100.0 | 0.0 | 100.0 |
| tRNA-Thr | 72 | 72 | 72 | 72 | 72 | 0.0 | 100.0 | 2.8 | 97.2 | 1.4 | 98.6 | 1.4 | 98.6 | 1.4 | 98.6 |
| tRNA-Pro | 70 | 70 | 70 | 70 | 70 | 2.9 | 97.1 | 1.4 | 98.6 | 4.5 | 95.7 | 2.9 | 97.1 | 0.0 | 100.0 |

**Supplementable Table 4** The spacer among the mitochondrial genes in the KOC, BSB, RCC-L, GF-L, RCC and GF

| **Genes** | **Numbers of the nucleotide spacer (bp)** | | | | | | **Sequences of the spacer (5，-3，),on the height strand** | | | | | |
| --- | --- | --- | --- | --- | --- | --- | --- | --- | --- | --- | --- | --- |
|  | GF-L | RCC-L | KOC | BSB | RCC | GF | GF-L | RCC-L | KOC | BSB | RCC | GF |
| tRNA-Leu and ND1 | 1 | 1 | 1 | 1 | 1 | 1 | T | T | T | C | T | T |
| ND1 and tRNA-Ile | 4 | 4 | 4 | 4 | 4 | 4 | CTAA | CTAA | TTAA | TTCA | CTAA | ACCC |
| tRNA-Gln and tRNA-Met | 1 | 1 | 2 | 1 | 1 | 1 | T | A | A | A | A | A |
| tRNA-Trp and tRNA-Ala | 2 | 2 | 2 | 1 | 2 | 2 | AT | AT | AT | A | AT | AT |
| tRNA-Ala and tRNA-Asn | 1 | 1 | 1 | 1 | 1 | 1 | A | A | A | A | A | A |
| tRNA-Asn and tRNA-Cys | 33 | 33 | 33 | 32 | 33 | 33 | CTTTTCCCGCCGTTTAA  CTCAGAAAGGCGGGAA | CTTTTCCCGCCGTTTAACTC  AGAAAGGCGGGAA | CTTTTCCCGCCGTTTAACTCAGTA  AGGCGGGAA | CTTTTCCCGCCTATGGCCTAGTAAGGCG  GGAA | CTTTTCCCGCCGTTTAACTCA  GAAAGGCGGGAA | CTTTTCCCGCCGTTTAACTCAGAAAGGCGGGAA |
| tRNA-Cys and tRNA-Tyr | 0 | 0 | 0 | 2 | 0 | 0 | ----- | ---- | ----- | GA | ---- | ----- |
| tRNA-Tyr and COI | 1 | 1 | 1 | 1 | 1 | 0 | T | T | T | T | T | ------ |
| tRNA-Ser and tRNA-Asp | 3 | 3 | 3 | 2 | 3 | 3 | CTA | CTA | CTA | CT | CTA | CTA |
| tRNA-Asp and CoII | 2 | 12 | 13 | 13 | 12 | 12 | AC | CACTTAAAATTA | ACCCAAAGACTTA | AGCTCAAGGCTTA | CACTTAAAATTA | CACTTAAAATTA |
| tRNA-Lys and ATPase 8 | 1 | 1 | 1 | 1 | 1 | 1 | A | A | A | A | A | A |
| tRNA-Ser and tRNA-Leu | 1 | 1 | 1 | 1 | 1 | 0 | C | C | C | C | C | ----- |
| tRNA-Leu and ND5 | 3 | 3 | 3 | 0 | 3 | 3 | AAA | AAA | AAA | ATG | AAA | AAA |
| ND6 and tRNA-Glu | 7 | 0 | 0 | 0 | 0 | 0 | AGGTCAT | ----- | ----- | ---- | ---- | ------ |
| tRNA-Glu and Cyt b | 5 | 5 | 5 | 4 | 5 | 5 | CATTA | CATTA | CACTA | CATA | CATTA | CATTA |
| Cyt b and tRNA-Thr | 0 | 0 | 0 | 4 | 0 | 0 | ---- | ----- | ---- | GCTT | ------ | ------ |

**Supplementable Table 5** The overlap of mitochondrial genome genes between KOC, BSB, RCC-L, GF-L, RCC and GF

| **Genes** | **Number of the nucleotide overlap (bp)** | | | | |  | **Sequences of the overlap (5’-3’on the height strand)** | | | | |  |
| --- | --- | --- | --- | --- | --- | --- | --- | --- | --- | --- | --- | --- |
|  | **GF-L** | **RCC-L** | **KOC** | **BSB** | **RCC** | **GF** | **GF-L** | **RCC-L** | **KOC** | **BSB** | **RCC** | **GF** |
| tRNA-Ile and tRNA-Gln | 2 | 2 | 2 | 2 | 2 | 2 | CT | CT | CT | TT | CT | TA |
| tRNA-Cys and tRNA-Tyr | 1 | 1 | 1 | 0 | 1 | 1 | T | T | T | ------- | T | T |
| ATPase 8 and ATPase 6 | 7 | 7 | 7 | 7 | 7 | 7 | ATGATAG | ATGATAG | ATGATAG | ATGCTAA | ATGATAG | ATGATAG |
| ND4L and ND4 | 7 | 7 | 7 | 7 | 7 | 7 | ATGCTAA | ATGCTAA | ATGCTAA | ATGCTAA | ATGCTAA | TACCCAC |
| ND5 and ND6 | 5 | 4 | 4 | 4 | 4 | 4 | TCTAA | CTAA | TTAA | TTAA | CTAA | CCAC |
| tRNA-Thr and tRNA-Pro | 1 | 1 | 1 | 1 | 1 | 1 | T | C | C | C | C | T |

**Supplementable Table 6** Nucleotide composition of different parts of the mitochondrial genome of KOC, BSB, RCC-L, GF-L, RCC and GF

| **mtDNA** | **GF-L** | | | | **RCC-L** | | | | **KOC** | | | | **BSB** | | | | **RCC** | | | | **GF** |  | |  |
| --- | --- | --- | --- | --- | --- | --- | --- | --- | --- | --- | --- | --- | --- | --- | --- | --- | --- | --- | --- | --- | --- | --- | --- | --- |
|  | **T%** | **C%** | **A%** | **G%** | **T%** | **C%** | **A%** | **G%** | **T%** | **C%** | **A%** | **G%** | **T%** | **C%** | **A%** | **G%** | **T%** | **C%** | **A%** | **G%** | **T% C%** | **A%** | **G%** | |
| **Complete** | **26.1** | **26.3** | **31.6** | **16.1** | **26.0** | **26.3** | **31.5** | **16.2** | **24.8** | **27.6** | **31.8** | **15.8** | **24.7** | **27.9** | **31.2** | **16.2** | **26.1** | **26.2** | **31.6** | **16.0** | **26.1 26.3** | **31.6** | | **16.0** |
| **13protein genes** | **28.1** | **26.9** | **29.7** | **15.3** | **28.1** | **26.8** | **29.6** | **15.4** | **26.6** | **28.4** | **29.8** | **15.2** | **26.6** | **28.7** | **29.0** | **15.6** | **28.3** | **26.7** | **29.7** | **15.3** | **28.2 26.8** | **29.7** | | **15.3** |
| **1st coden** | **21.4** | **26.2** | **26.7** | **25.7** | **21.4** | **25.8** | **26.6** | **26.1** | **20.4** | **26.7** | **26.8** | **26.1** | **20.5** | **26.5** | **27.1** | **26.0** | **21.3** | **25.9** | **26.7** | **26.1** | **21.3 25.9** | **26.7** | | **26.1** |
| **2nd coden** | **40.7** | **27.0** | **18.5** | **13.7** | **40.7** | **27.3** | **18.3** | **13.7** | **40.7** | **27.3** | **18.3** | **13.8** | **40.6** | **27.4** | **18.3** | **13.7** | **40.8** | **27.1** | **18.3** | **13.7** | **40.7 27.2** | **18.3** | | **13.7** |
| **3 rd coden** | **22.1** | **27.4** | **44.1** | **6.4** | **22.3** | **27.4** | **43.9** | **6.4** | **18.7** | **31.3** | **44.3** | **5.7** | **18.8** | **32.2** | **41.8** | **7.1** | **22.7** | **27.2** | **44.1** | **6.0** | **22.6 27.2** | **44.1** | | **6.0** |
| **2 rRNA genes** | **20.2** | **24.1** | **34.6** | **24.1** | **20.2** | **24.1** | **34.6** | **21.1** | **19.3** | **25.0** | **35.0** | **20.7** | **19.7** | **24.5** | **34.5** | **21.3** | **20.2** | **24.1** | **34.7** | **21.0** | **20.2 24.0** | **34.6** | | **21.1** |
| **22 tRNA genes** | **25.1** | **24.3** | **30.7** | **19.9** | **25.3** | **24.3** | **30.2** | **20.2** | **25.5** | **23.6** | **30.5** | **20.4** | **25.5** | **24.7** | **30.1** | **19.8** | **25.1** | **24.2** | **30.6** | **20.1** | **27.1 20.7** | **28.6** | | **23.6** |
| **D-loop region** | **32.7** | **20.5** | **32.4** | **14.5** | **31.8** | **21.5** | **32.7** | **14.0** | **33.0** | **19.7** | **33.3** | **13.9** | **30.5** | **21.8** | **33.3** | **14.4** | **32.5** | **20.8** | **32.5** | **14.2** | **32.3 20.9** | **32.5** | | **14.2** |

**Supplementable Figure 1**

Phylogenetic relationships among KOC, BSB, RCC-L, GF-L, RCC, GF and Danio rerio based on entire mtDNA by using IQ-tree

**
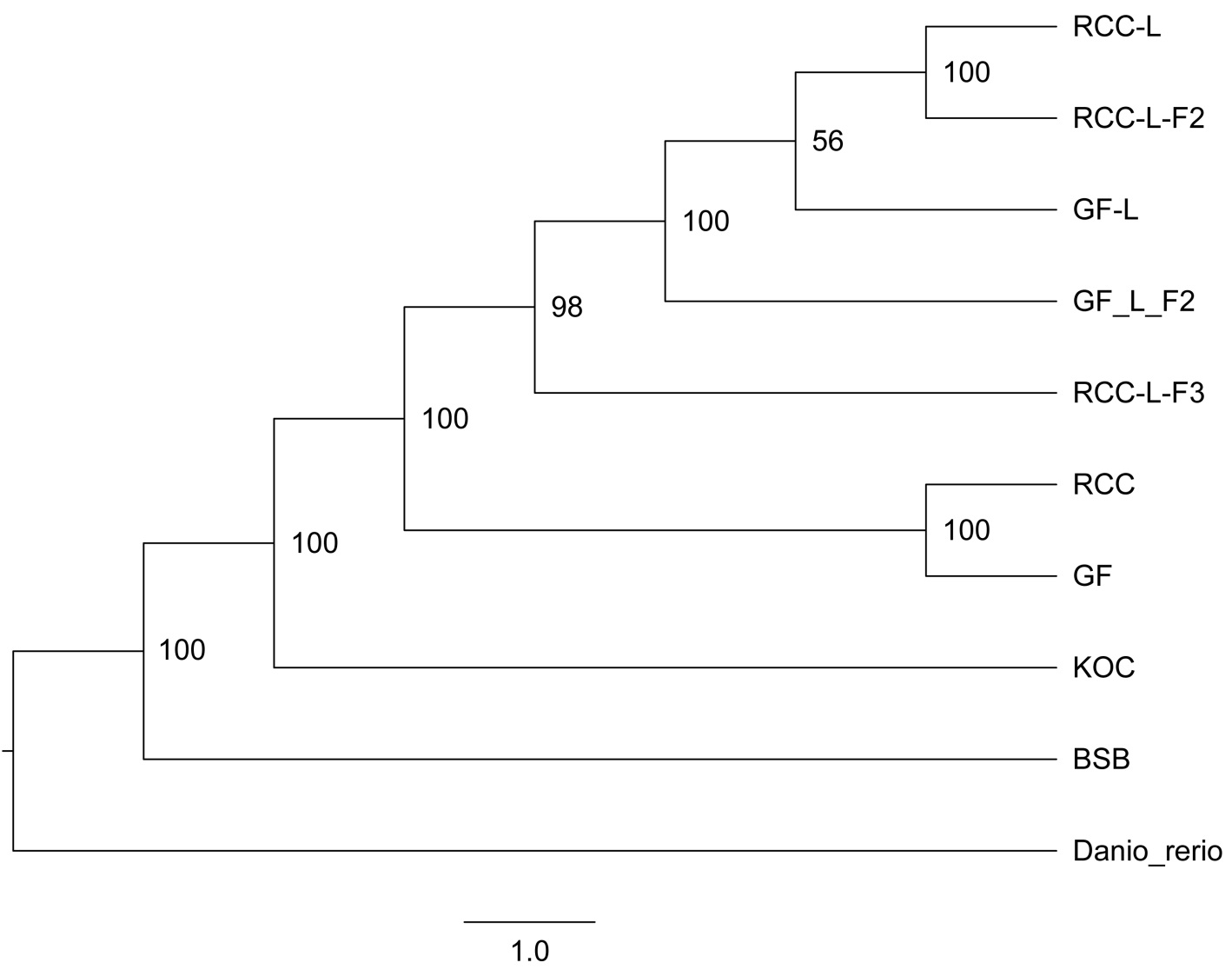
**


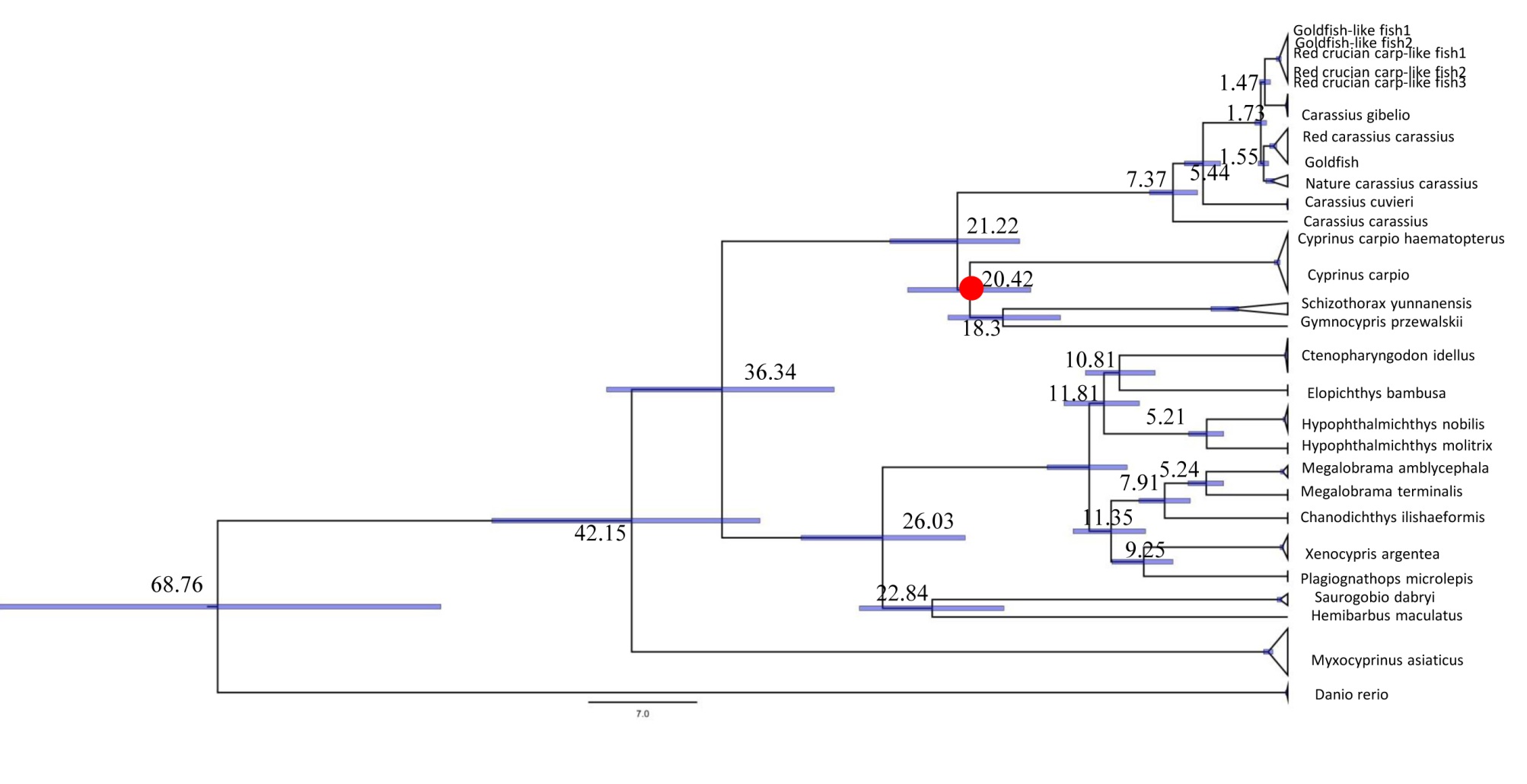


**Supplementable Figure 2**

Dated phylogeny using one calibration point [red dots indicate the time-calibration marker (Wang et al., 2016; Wu et al., 2020)] to estimate the divergence of *Carassius*. Node ages are shown together with 95% highest posterior density bars indicating a range of age estimates
